# Aging Narratives over 210 years (1810-2019)

**DOI:** 10.1093/geronb/gbaa222

**Published:** 2020-12-10

**Authors:** Reuben Ng, Ting Yu Joanne Chow

**Affiliations:** 1 Lee Kuan Yew School of Public Policy, National University of Singapore, Singapore, Singapore; 2 Lloyd’s Register Institute for the Public Understanding of Risk, National University of Singapore, Singapore, Innovation 4.0, 3 Research Link, Singapore

**Keywords:** Ageism, media portrayals of aging, age stereotypes, age discrimination, medicalization of aging, psychomics, social gerontology, social ostracism, historical analysis

## Abstract

**Objectives:**

The World Health Organization launched a recent global campaign to combat ageism, citing its ubiquity and insidious threat to health. The historical context that promoted this pernicious threat is understudied, and such studies lay the critical foundation for designing societal-level campaigns to combat it. We analyzed the trend and content of aging narratives over 210 years across multiple genres—newspaper, magazines, fiction, non-fiction books; and modelled the predictors of the observed trend.

**Methods:**

A 600-million-word-dataset was created from the Corpus-of-Historical-American-English and the Corpus-of-Contemporary-American-English to form the largest structured historical corpus with over 150,000 texts from multiple genres. Computational linguistics and statistical techniques were applied to study the trend, content, and predictors of aging narratives.

**Results:**

Aging narratives have become more negative, in a linear fashion (p=.003), over 210 years. There are distinct shifts: From uplifting narratives of heroism and kinship in the 1800s to darker tones of illness, death, and burden in the 1900s across newspapers, magazines, and non-fiction books. Fiction defied this trend by portraying older adults positively through romantic courtship and war heroism. Significant predictors of ageism over 210 years are the medicalization of aging, loss of status, warmth, competence, and social ostracism.

**Discussion:**

Though it is unrealistic to reverse the course of ageism, its declining trajectory can be ameliorated. Our unprecedented study lay the groundwork for a societal level campaign to tackle ageism. The need to act is more pressing given the Covid-19 pandemic where older adults are constantly portrayed as vulnerable.

Ageism—the discrimination of older adults based on their age (Butler, 1969)—has undergone a nuanced progression throughout the last two centuries. Pre-nineteenth century, older adults were perceived as esteemed and authoritative—particularly as fewer of them lived to old age—however, post-nineteenth century, shifting trends in modernity resulted in aging being framed as a social problem than a natural process (Applewhite, 2019). Since then, ageism has had a long history of intervention strategies to counteract it. Many of these mitigation efforts build on the pioneering efforts of Butler, whose legacy of reframing ageism added to the gerontologic repertoire of concepts like productive and successful aging; and in enhancing long-term care through his founding of research institutions like the National Institute on Aging (Achenbaum, 2014). Institutionally, groups like the AARP and the Grey Panthers have been instrumental in pushing congressional enactment of the Age Discrimination in Employment Act in 1967; later amended in 1986 to abolish mandatory retirement of older adults (Issacharoff & Harris, 1997). These efforts have spurred ageism into society's consciousness, especially in the latter half of the 20th century.

More recently, campaigns to combat ageism have continued to persist in the same vein. At the individual level, there have been significant progress in designing inter-generational programs to reduce ageism (Lytle et al., 2020). These programs are predicated on Palmore's (1977) landmark 'facts on aging' quiz (updated by Breytspraak & Badura, 2015) that identified the content of negative age stereotypes, and increasingly used in medical education (Unwin et al., 2008). At the societal level, campaigns to combat the overwhelming negative portrayals of older adults in the media are few, with the most recent 'Disrupt Aging Collection' by AARP/Getty that presented realistic and positive images of aging (AARP, 2020). In 2016, 194 World Health Organization member states called on the international organization to develop a global campaign to combat ageism, citing its alarming ubiquity and insidious threat to health (Officer & de la Fuente-Núñez, 2018)—age stereotypes can be internalized across one's lifespan and detrimentally affects one's health when it becomes self-relevant (Lakra et al., 2012; Levy, 2009; Ng et al., 2016) and is linked to poorer health, and increased mortality (Chang et al., 2020; Ng, Allore et al., 2020).

The effectiveness of such laudable campaigns depends on the ability to target the deep-seated drivers of ageism that have percolated over the last 200 years—a time period that traditional methods cannot measure. Against this historical backdrop, we analyze how older adults have been portrayed in various media genres across these 200 years. Recent grants by the National Endowment for the Humanities (NEH) and the National Science Foundation (NSF) provided unprecedented linguistic datasets, and innovative scholars have created 'Psychomics'—a methodology to analyze societal stereotypes and narratives using high throughput sematic data (Ng et al., 2015; Ng & Lim, 2020).

We leverage these developments to study aging narratives to fulfill three research objectives—and test three hypotheses: First, we analyze the trend of aging narratives over 21 decades (1810-2019) by integrating the Corpus of Historical American English (COHA; 1810-2009), and the Corpus of Contemporary English (COCA; 2010-2019) to form the largest historical corpus of American English—600 million words—with over 150,000 texts from newspapers, magazines, fiction, non-fiction books. We hypothesize that aging narratives have become more negative over 21 decades (Hypothesis 1). Second, we analyze the narratives underlying the trend, specifically, the evolution from the $19^{th}$ to $20^{th}$ centuries, across multiple genres—newspapers, magazines, fiction, and non-fiction books. We hypothesize that the content of aging narratives in the $19^{th}$ century will contain a majority of positive themes while those in the $20^{th}$ century will contain a majority of negative themes (Hypothesis 2). Third, we test multiple hypotheses of the declining trend in aging narratives over 200 years: loss of status, warmth, competence, social ostracism, and the medicalization of aging (Hypothesis 3). Hypothesis 3 expands the literature on sociological drivers of aging narratives beyond previous studies (e.g., Ng et al., 2015) that focused on a singular sociological variable (medicalization of aging), and population aging. Interestingly, medicalization of aging was significantly associated with age stereotypes, after adjusting for population aging—suggesting that sociological variables are equally, if not more, important when studying the historical underpinnings for aging perceptions. Against this background, we chose to delve deeper into the sociological variables that are linked to aging narratives over 21 decades.

Our study is significant in three ways. Conceptually, this is one of the first known studies to analyze the content of aging narratives across 210 years in a massive and comprehensive historical corpus spanning multiple genres. Previous studies, though comparable in historical horizon (Ng et al., 2015), analyzed only stereotypic value (positive vs negative) but lacked the nuances and depth provided by our study. Such color and texture, provided by narratives, are crucial to distilling the sociological underpinnings of ageism across 210 years—our study's second key contribution. Practically, the study lays the groundwork for an effective societal campaign to counteract ageism. Of broader significance, we created an unprecedented platform for policy makers and scholars to study societal narratives that compliments traditional methods of focus groups and surveys.

**Historical Content and Measurement of Aging Narratives**

We synthesize the extant literature on the historical content and measurement of aging narratives to highlight the need for more rigorous datasets and methods that our study provides. Scholars painted a declining image of the old person: from the pedestal of prestige in the early 19th century to the plains of negativity in recent decades. Archival evidence from selected 19th century sermons suggested that growing old was a natural process to be celebrated (Cole, 1993). The advent of industrialization—which promoted speed and efficiency as hallmarks of success (Taylor, 1911)— reduced the esteem given to the experience of the older population and instead magnified the physical shortcomings of old age, spawning an industry devoted to reversing them.

A vast library of scholarly work have been devoted to investigating perceptions through situational judgement of tests (e.g., Ng & Rayner, 2010), and surveys that found a high prevalence of ageism (Palmore, 2001; McGuire et al., 2008), even among healthcare workers (Makris et al., 2015) where older adults were commonly perceived as frail and weak. Young people harbor conflicted and negative emotions about growing old (Berger, 2017). Furthermore, it remains common, albeit erroneously reductionist, to simply generalize old people as a homogenous group without considering that they are just as diverse in gender, ethnicity, and profession—and the sociological literature have worked on framing these forms of prejudice (Blaikie, 1999; Gullette, 2004).

Ageism also manifests in many qualitative studies: Birthday cards hinting that an older adult's best days are behind them, a booming beauty industry designed to hide signs of aging, healthcare practitioners harboring preferential bias against older adults (Nelson, 2016). Advertisements in the early 1900s started to popularize the scientific management of aging and peddled "fixes" for age-associated changes such as balding (Andrews, 1999). These indirect methods are helpful to understand aging narratives, as cultivation theory posits that different forms of media reflect societal perceptions they seek to portray (Gerbner et al., 2002). However, they are single-sourced, anecdotal, and run the risk of cherry-picked sources to support a scientific agenda (Mautner 2005). Importantly, single-sourced studies at different snapshots in history do not provide strong evidence for a targeted media campaign at a societal level. This is likely due to the lack of suitable large-scale datasets and methods to interrogate them.

Recent advancements in corpus linguistics and quantitative social sciences enabled scholars to go beyond single sources to leverage multiple genres. For example, Mason and colleagues (2015) used the Google Book Ngram to examine how gender-based terms ‘old man’ and ‘old woman’ changed in frequency over time. They found that ‘old man’ was used three times more than ‘old woman’ suggesting a gender bias in books. Their analysis provided only a glimpse to the frequency of conversations in digitized books but not its content. Ng and colleagues (2015) took a more rigorous approach by collating a comprehensive list of 11 synonyms of ‘older adult’ over 200 years and analysed their descriptors for stereotypic content (negative/positive). While both studies made noted advancements in the historical study of societal stereotypes of aging, they fall short in delineating the underlying content of these stereotypes. Our study makes an important contribution by unpacking the richness and nuances of aging narratives across 21 decades. These insights provide a deeper appreciation for the historical context and sociological drivers that have percolated over two centuries to produce the current societal embodiment of the older person.

### Methods

#### Dataset

Our 600-million-word corpus spanning 21 decades from 1810 to 2019 was created by integrating the respective decades from the Corpus of Historical American English (COHA; 1810-2009) and the Corpus of Contemporary American English (COCA; 2010-2019). This is the first known integration of both prominent corpora to form the largest structured historical English corpus.

COHA is 100 times larger than other equivalent historical corpora and used by major studies to test historical shifts in narratives (Ng et al., 2015). It consists of more than 100,000 sources including popular magazines, newspapers, fiction, and non-fiction books. Their sub-genres are equally represented in this corpus from 1810 to 2009. This diverse and well-balanced corpus captures “real world” cultural and social changes in the U.S. (Davies, 2012).

It is structured for frequency analysis of specific keywords and the context of the word used in each decade, empowering researchers to capture and understand dynamics of societal narratives. The COCA is the largest, balanced corpus of contemporary American English. The corpus contains more than one billion words of text, including 20 million words each year from 1990, and it is equally divided among spoken, fiction, popular magazines, newspapers, and academic texts. The even distribution of various sources gives the corpus its balance. The corpus is also updated every six to nine months, therefore serving as a unique record of linguistic changes in American English (Davies, 2009). According to Cultivation Theory (Gerbner et al., 2002) different forms of media within the corpus—newspapers, magazines, fiction, non-fiction books—reflect societal perceptions of the respective eras and provide an extraordinary platform to study aging narratives.

**Measurement of Aging Narratives, Predictors, and Analytic Strategy**

All synonyms of "older adult" from 1810-2019 were compiled from the Historical Thesaurus of the Oxford English Dictionary (Kay et al., 2009), and the Oxford Thesaurus (Waite, 2006), resulting in 18 synonyms. Four (emerit, gerontic, through-old, old aged) did not show up in the corpus; three (eldern, well-aged, passing old) appeared less than 10 times in 210 years and did not refer to an older adult. Eleven target synonyms were retained for analysis (e.g., aged, elderly, old people, senior citizen, older adult, golden ager), and the top 100 words that co-occur most frequently (collocates) with each of these 11 synonyms were compiled per decade for 210 years, with the following inclusion criteria: (a) Lexical Proximity: collocate present within four words prior or after the target synonym; (b) Relevant context: collocate referred to specifically to an old person (checked by two raters); (c) Mutual Information Score of three and above—indicating semantic bonding where the collocate has a stronger association with the respective synonym than other words in the corpus (Church &

Hanks, 1989). This is an innovative application of concordance analysis, used in computational linguistics to study narrative evolution (Ng & Lim, 2020).

To test Hypothesis 1, each collocate that met the study criteria (N=14,678) was rated on a scale from 1 (very positive) to 5 (very negative), a method found to be valid and reliable to measure age-stereotype associated words (Levy & Langer, 1994). The methodology is consistent with previous corpus-based studies (e.g., Ng et al., 2015; Ng & Lim, 2020), and is predicated on the priming literature (e.g., Levy, 2009) showing that repeated associations of negative words with older adults, in close lexical proximity, increases implicit ageism. For example, very negative collocates were rated 1 (e.g. *abuse, frail*), neutral collocates were rated 3 (e.g. *apartment, armchair*), and very positive collocates were rated 5 (e.g. *affectionate, wise*). The inter-rater reliability using Cronbach's alpha was .984 (95% CI: .980, .988) for the scoring method. For every synonym per decade, we calculated a mean score that was subsequently weighted (by the number of times the synonym appeared in that decade) to yield a Cumulative Aging Narrative Score (CANS) for the respective decade, and we conducted a trend analysis of the respective aging narrative score across 21 decades.

To test Hypothesis 2, we ran a Latent Dirichlet Allocation (LDA)—a well-established machine learning technique for topic modelling—on the collocates of the 19th and 20th century, respectively. Hypothesis 3 was tested by a linear mixed model with the cumulative aging narrative score as the outcome, decade as the random factor and fixed factors—loss of status, warmth, competence, social ostracism, and the medicalization of aging, controlling for covariates. *Loss of Status* is measured by "does this collocate describe the power or position of this person?" on the following scale: 1 (low status), 2 (neutral), 3 (high status). Examples include 'lowly' rated as '1', 'military-looking' rated as '3'. The Stereotype Content Model proposed that social perceptions form along the dimensions of cold/warm and incompetent/competent (Fiske et al., 2002). Against this background, *Loss of Warmth* is measured by “does this collocate describe the good/bad intentions of a person?” on the following 5-point scale from 1 (very cold) to 5 (very warm). For example, ‘sincere’ and ‘trustworthy’ will be rated ‘very warm’ while ‘aloof’ and ‘unfriendly’ will be rated ‘very cold’. *Loss of Competence* is measured by “does this collocate describe the ability of a person?” on the following 5-point scale from 1 (very incompetent) to 5 (very competent). For example, ‘smart’ will be rated ‘very competent’ while ‘simple-minded’ will be rated ‘very incompetent’. *Medicalization of Aging* is measured by “does this collocate describe or relate to the physical health of a person?” on a binary scale of 0 (No) and 1 (Yes). Sample words that are rated ‘1’ are ‘sick’. *Social Ostracism* is measured by “does this collocate describe a person’s group membership?” on a binary scale of 0 (No) and 1 (Yes). The inter-rater reliability using Cronbach’s alpha for all the aforementioned variables ranged from .982 to .991 (95% CI: .979, .994).

## Results

### Societal Sentiments of Older Adults over 21 Decades

As hypothesized, Cumulative Aging Narratives Score (CANS) declined over 21 decades in a linear fashion (Figure 1), indicating that sentiments towards older adults have become more negative ($\beta$ = -0.0068 $p$ = 0.0029). Neither the quadratic ($\beta$ = -0.00026 $p$ = 0.49) or the cubic ($\beta$ = -0.00009 $p$ = 0.19) models reached significance. The unstandardized intercept of the linear trend was 3.08 (95% *CI*: 3.05, 3.10, $p$ < 0.00001) indicating that aging narratives were positive in the early 1800s. However, with each passing decade between 1810 to 2019, there was a 0.142-unit (95% *CI*: -0.101, -0.185) decrease. The proportion of decades with CANS above the neutral threshold fell from 66.7% in the 1800s to 30% in 1900s. Taken together, this suggests that aging narratives have become more negative over 210 years, providing support for Hypothesis 1 (Figure 1).

**Shift in Aging Narratives over Two Centuries across Genres**

**Summary Insights.** Latent Dirichlet Allocation (LDA) is a natural language processing method that analyzed topic clusters within respective genres. For each of our four genres, we generated a list of topics for the 1800s and 1900s to track the shift in aging narratives across centuries. Overall, we found distinct shifts from uplifting narratives of heroism and kinship in the 1800s to darker tones of illness, death, and burden in the 1900s across all genres of the largest historical corpus of 600-million-words—providing support for Hypothesis 2. Newspapers focused on the drama of family life in the 1800s and transited to more serious topics such as social welfare, disability and chronic care across the 1900s. Magazines and non-fiction books followed the same trajectory from an 1800s focus on family, war heroism, love and royalty to the gravity of illness and the institutions of care in the 1900s. Fiction evidenced a more neutral trajectory with 1800s themes of war heroism and the daily grind to 1900s themes of showbiz and retirement homes. Courtship and romance remained consistent themes throughout 21 decades in fiction.

**Newspapers 1800s.** The biggest topic (Topic 1) is marital affairs, and likely about the legalities behind marriage (wife, endow [i.e. 'dowry'], furnish, discuss); Topic 2 related to marital status (widow, bride). Another pertinent topic is courtroom proceedings: Topic 3 contained collocates about the judicial branch (solicitor, judge, order); Topic 4 about official documents (paper, report) and official affairs (charge, chamber). Another salient topic is familial affairs: kinship terms are employed (daughter, relative) in Topic 5; and peppered throughout the dataset (wife, bride, husband, son, brother).

**Newspapers 1900s.** The topic of societal issues appears in Topic 1, with the use of words that convey a reliance (depend, fear) on assistance (aid, plan, official); and in Topic 2 on crime (police, fight, assist); and Topic 3 on dissatisfaction with societal problems like the cost of living (cost, income) and external stressors (attack, force). The remaining topics are centered on public health issues and public welfare, where Topic 4 contains words about receiving medical attention (medical, care, health, hospital); Topic 5 about institutional (federal, senate) spending on welfare (spend, public, welfare); Topic 6 on monetary aid (tax, support, pension) for older adults in need (receive, apply); Topic 7 on societal support (benefit, education, build) for the less fortunate (poor, disabled).

These prevalent topics suggest that older-adult-related news in the 1800s had a focus on family and the law. Conversely, 1990s news topics are centered on public health and societal issues.

**Magazines 1800s**. Topics encompass war and family honor: Topic 1 covers aspirations (love, dream) in the war; Topic 2 about post-mortem honor (death, honor, present, receive) and words of veneration (venerable, memory); Topic 5 of national history (respect, history) and family (lady, maternal, son, boy). The topic of illness in the family is in Topic 3, with words about death (die) and sickness (infirm, fall, care) in the family (daughter, brother); and Topic 4, where sickness (sick) is present in the family (relative).

**Magazines 1900s.** The topic of war and family honor is present in Topic 2 (war, officer), and on family (wife, husband, uncle); Topic 5 in honoring a generation of nation-builders (retire, build, nation). Topic 1 contains the topic of family, with kinship terms (daughter, parent, sister, brother) and motherhood-related words (maternal, born, baby); Topic 6 about growing up (boy, youth, grow). Another prevailing topic is of death and illness: Topic 3 contains words about medical welfare (care, health, medical, hospital); Topic 4 on imminent death (die, death, nurse, visit); Topic 7 about severe illness (sick, severe, kill, patient).

While the key older-adult-related topics in magazines are similar (i.e. both 1800s and 1900s have similar topics like war and family honor, and illness), the topic of death and illness in the 1900s contains more severe collocates (die, death, kill, severe) and collocates about public health (hospital, medical, nurse) compared to the 1800s; thus suggesting a subtle shift toward negativity.

**Non-Fiction 1800s.** The topic of family and marriage is present in Topic 1, with father-son dyadic relationship terms (son, paternal) and the cycle of life and death (die, born); Topic 3 on marriage (marry, daughter); Topic 5 about the monarchy (king) and familial terms (brother, sister, grandchildren, maternal). The topic of death and illness is present in Topic 2, on sickness (sick, infirm) in the family (parent, wife, dear); and in Topic 4, with words connoting to mortality (kill, death) in the family (boy, daughter) and post-mortem arrangements (afterward, church, fortune, remember).

**Non-fiction 1900s**. The topic of death and illness is common: Topic 1 relates to illnesses (ill, severe) among the population (population, person, girl, son); Topic 2 on medical care (care, patient, death); Topic 3 on the medical circumstances of the less-fortunate (die, poor); Topic 4 on sickness (disease, death, health); and Topic 5 on public health (hospital, assist, public).

In sum, older-adult-related topics in the 1800s had a family-heavy focus on genealogy and kinship terms; and took a darker tone in the 1900s with its most prominent topics being entirely focused on death, illness, and health.

**Fiction 1800s.** Fiction in the 1800s revolves around courtship and romance: Topic 1 contains words about women (girl, lady, daughter, wife); Topic 3 on romance (love, heart, care, dear); Topic 4 about courtship and physical appearance (lady, gentleman, beautiful, wear, dress). Other topics include heroism and wartime stories: Topic 2 contains words connoting to veneration for the departed (respect, dead, person); Topic 5 about honoring veterans (remember, war) and appreciating peacetime (present, happy, wonder, rest). Another topic is daily interaction: T6 contains everyday exchanges (smile, reply) and Topic 7 contains words on the indoor setting and activities (chair, seat, read).

**Fiction 1900s.** The topic of daily interaction is found in Topic 1, related to the indoor setting and household furniture (chair, bed, table, kitchen, window); Topic 3 about feeling happiness (smile, happy); Topic 6 contains quotidian tasks (open, carry, door). The topic of death is present in Topic 2, with words about death (die, dead) in the family (son, cousin, wife, sister). The topic of showbiz is present in Topic 4, with words about filming equipment (camera, light) and stage directions (speak, walk, enter, toward, step). The topic of romance is present in T5, about couples (gentleman, dear) interacting (laugh, grow, stay); Topic 7 about beloved individuals (love, lady, heart).

Both the 1800s and 1900s contain themes on entertainment (showbiz, romance, courtship) and daily life (indoor setting, daily interactions). Thus, fiction was the only genre of texts that did not present any notable negative changes in the way the older adults have been portrayed.

**Factors Associated with Declining Trend of Aging Narratives across 210 Years**

We used linear mixed models to analyze predictors of the declining trend of aging narratives and found support for hypothesis 3. High status descriptors of older adults have decreased, indicating the diminishing status of older adults, $\beta = 0.68$, $p = 0.003$; physical health descriptors have increased, indicating the medicalization of aging, $\beta = -1.26$, $p = 0.013$; social ostracism descriptors have increased, $\beta = -0.79$, $p = 0.033$; warm ($\beta = 0.37$, $p = 0.043$) and competent ($\beta = 0.44$, $p = 0.047$) descriptors have decreased significantly.

### Discussion

This is one of the first known studies to analyze the historical content of societal ageism over an unprecedented time horizon of 210 years from 1810-2019. This is achieved through the innovative merger of well-used corpora to create the largest structured corpus of historical English containing 600 million words across multiple genres—and the creative applications of computational linguistics and machine learning to the study of societal narratives (Ng et al., 2015). The key findings are consistent, though no less alarming: Aging narratives have steadily declined in a linear fashion over 21 decades. Across multiple genres—newspapers, magazines, and non-fiction books—uplifting narratives of heroism, honor, and kinship in the 1800s have evolved into darker themes of disability and death in the 1900s. The increasing prevalence of ageism across 210 years, according to cultivation theory, are instructive of societal narratives as the media reflect societal narratives they seek to portray (Gerbner et al., 2002).

Our study made three important contributions. First, our findings lend credence to existing scholarship—albeit mostly on single sources (e.g., art, ethnography or advertisements)—that older adults, since the late 1800s, were largely viewed as the dominant occupants of almshouses who were often described as deserted by their children and too infirm to work (Haber, 1993). While the early 1800s accentuated the positive image of older adults by featuring them as "remarkably ebullient, regardless of their social condition and economic status", the late 1800s to early 1900s saw a decline in their status with a majority portrayed as "among the other deserving poor in an almshouse" (Achenbaum, 1993). Policy debates have increasingly portrayed older adults as physically vulnerable who require social welfare (Rowles, 1993). Beside ageism, we also observed adjacent 'isms' such as sexism, reflected in the gender imbalance in themes where male-related words occur with wartime honor (venerable, honor, war, officer), and all female-related words revolve around the family and their relationship status (bride, wife, maternal, widow)—this underscores the notion of invisibility of older women who are sidelined and mentioned only in relation to the family unit (Woodward, 1999), and echoes Mason and colleague's (2015) finding that 'older men' appeared three times more than 'older women' in books. Other themes of racism and classism may also exist, and future studies should explore these complimentary phenomena.

Second, our study contributed to understanding the historical underpinnings of societal ageism. Ng and colleagues' (2015) historic study found that age stereotypes became more negative in a linear fashion: they were positive from 1810 to 1870, neutral in 1880 and negative from 1890 to

2009. Though the study added an important historical perspective to a crowded field of cross-sectional survey studies (e.g., Fernandez et al., 2015; Heidekrueger et al., 2017), it was focused mainly on the stereotypic value—positive/negative. Aging narratives over two centuries are richer, deeper and more complex than a single dichotomy (positive/negative) can capture. Moreover, there would be interesting differences across genres that Ng's (2015) study did not explore. Our study made a significant extension by exploring the rich content of aging narratives across multiple genres—newspapers, magazines, fiction, non-fiction books—over 210 years (we updated the dataset with one more decade of data, 2010-2019). The increasing negativity of aging narratives were found in newspapers, magazines, non-fiction books, though fiction defied this trend—interestingly, portraying older adults as war heroes and romantics across 210 years.

In addition, our study expanded the sociological drivers of the negative aging narratives beyond the single sociological variable—medicalization of aging—that Ng (2015) tested, after adjusting for demographics (population aging). A single attribute cannot capture the sheer complexity of societal factors that influence aging narratives over two centuries. Our study attempted to capture this complexity by testing five sociological variables (including the medicalization of aging) that emerged as independent predictors of age stereotypes, albeit in cross-sectional survey studies. To this end, we found that the diminishing status of older adults, loss of warmth, loss of competence, social ostracism and the medicalization of aging contributed to the increasing negativity of aging narratives over 21 decades. This cocktail of sociological drivers provides a guiding framework for focus areas of anti-ageism campaigns: Portray older adults as warm, competent, and socially engaged. Future campaigns should take note of these nuances and focus on these behavioral attributes, in addition to debunking physical stereotypes such as frailty.

While we sought to circumvent the weaknesses of other studies by providing a multi-sourced platform of 600 million words, this study is not without weaknesses. We did not include social media in our corpus as the genre came to being only in the last 20 years. Regardless, this is a significant weakness as ageism cannot be compared across media genres; furthermore, social media has popularized disparaging phrases like '*ok boomer*', contributing to intensifying intergenerational tensions (Meisner, 2020). A future iteration of the study should include social media, for comparative analysis of contemporary genres, to facilitate advocacy campaigns. Additionally, as this study focused on English-based texts throughout 210 years, we acknowledge that sources from other languages and cultures may yield different insights. Future studies could extent the linguistic scope.

**Conclusion**

Though it is unrealistic to reverse the course of ageism, its declining trajectory can be ameliorated. Our unprecedented study provides ideas for a societal level campaign that esteemed institutions like Gerontological Society of America (GSA) and the AARP should front, vigorously. The need to act is more pressing given the Covid-19 pandemic where older adults are constantly portrayed as the vulnerable and a high-risk group (Ng et al., 2021). If unchecked, ageism will exert a greater toll on mortality, healthcare cost and disability (Ng, Lim et al., 2020). We hope to have provided the sociological intelligence for designing interventions to tackle societal ageism—one of our generation's most insidious threats.

| Genre | 1800s Narratives | 1900s Narratives |
|---|---|---|
| Newspapers | Marriage<br>Family life<br>Courtroom proceedings | Chronic conditions<br>Social welfare<br>Disability |
| Magazines | Family honor<br>War heroism<br>Love | Death<br>Caregiving<br>Nursing homes |
| Non-fiction books | Family relationships<br>Marriage<br>Royal families | Death<br>Illness<br>Healthcare |
| Fiction books | Courtship and romance<br>War heroism<br>Daily grind | Showbiz<br>Retirement homes<br>Courtship and romance |

**Figure 1.** Cumulative Aging Narrative Score (CANS), depicting sentiments toward older adults, have declined over 210 years from 1810 to 2019. There are distinct shifts: From uplifting narratives of heroism and kinship in the 1800s to darker themes of illness, death and burden in the 1900s across newspapers, magazines and non-fiction books. Fiction defied this trend by portraying elders positively through romantic courtship, war heroism and retirement homes. Dots represent the CANS score in the respective decade and green shading represents the 95% confidence band.
